# Coronary angiography in acute ischemic stroke patients: frequency and determinants of pathological findings in a multicenter cohort study

**DOI:** 10.1007/s00415-022-11001-5

**Published:** 2022-02-19

**Authors:** Simon Litmeier, Thomas R. Meinel, Regina von Rennenberg, Joachim U. Kniepert, Heinrich J. Audebert, Matthias Endres, Simon Jung, Jan F. Scheitz, Christian H. Nolte

**Affiliations:** 1 Klinik und Hochschulambulanz für Neurologie, Charité - Universitätsmedizin Berlin, Corporate Member of Freie Universität Berlin, Humboldt-Universität zu Berlin, and Berlin Institute of Health (BIH), Berlin, Germany; 2 German Center for Neurodegenerative Diseases (DZNE), Partner Site Berlin, Berlin, Germany; 3 Center for Stroke Research Berlin (CSB), Charité-Universitätsmedizin Berlin, Berlin, Germany; 4 Department of Neurology, Inselspital, University Hospital Bern, University of Bern, Stroke Research Center Bern, Bern, Switzerland; 5 Berlin Institute of Health (BIH) at Charité-Universitätsmedizin Berlin, Berlin, Germany; 6 German Center for Cardiovascular Research (DZHK), Partner Site Berlin, Berlin, Germany; 7 ExcellenceCluster NeuroCure, Charité-Universitätsmedizin Berlin, Berlin, Germany

**Keywords:** Heart and brain axis, Acute ischemic stroke, Myocardial injury, Stroke-heart-syndrome

## Abstract

**Background:**

Myocardial injury as indicated by cardiac troponin elevation is associated with poor prognosis in acute stroke patients. Coronary angiography (CAG) is the diagnostic gold-standard to rule-out underlying obstructive coronary artery disease (CAD) in these patients. However, weighing risks and benefits of coronary angiography (CAG) against each other is particularly challenging, because stroke patients undergoing CAG may have a higher risk for secondary intracranial bleeding. Current guidelines remain vague. Thus, the aim of this study was to analyze frequency of pathological findings of CAG and associated clinical factors.

**Methods:**

We analyzed indications and frequency of CAG performed in acute ischemic stroke patients in clinical routine in two European tertiary care hospitals from 2011 to 2018. All data were obtained retrospectively. Multiple logistic regression analyses were performed to identify variables associated with absence of obstructive coronary artery disease defined as presence of at least one coronary vessel stenosis ≥ 50%.

**Results:**

A total of 139 AIS patients underwent CAG. Frequent indications for CAG were suspected acute coronary syndrome (*N* = 114) or scheduled cardiac surgery (*N* = 25). Acute coronary stenting was applied in 51/139 patients. Among patients with suspected acute coronary syndrome, no obstructive CAD was found in 27/114 patients. Absence of obstructive CAD was associated with insular cortex lesions, no clinical symptoms for ACS, less than three cardiovascular risk factors, younger age and normal wall motion.

**Conclusion:**

Several variables suggest absence of CAD in AIS patients and may help in clinical decision making in stroke patients with myocardial injury.

**Supplementary Information:**

The online version contains supplementary material available at 10.1007/s00415-022-11001-5.

## Background

Patients with acute ischemic stroke (AIS) and myocardial infarction (MI) share the same risk factors [1]. The risk for AIS patients to subsequently suffer a MI is high and vice versa [2]. One out of five AIS patients will experience a serious cardiac adverse event within the first 3 months after stroke with cardiac complications peaking at day 2–3 after the cerebral event [3]. Current American Heart Association/American Stroke Association treatment guidelines recommend the assessment of cardiac troponins (cTn) in AIS patients because of the close association between stroke and cardiac abnormalities [4]. Up to 60% of AIS patients have high-sensitivity cardiac Troponin T (hs-TnT) levels above the upper reference limit defining myocardial injury and approximately 15% of patients have hs-TnT levels above the ‘rule-in’ threshold for MI [5]. However, on an individual patient level, it remains challenging to differentiate acute coronary syndrome (ACS) from other causes of myocardial injury [6]. Invasive coronary angiography (CAG) remains the gold standard to assess the coronary vessel status and diagnose ACS [7]. However, CAG is associated with significant periprocedural risk [8]. Periprocedural heparin increases the risk of hemorrhagic transformation of vulnerable ischemic brain tissue and ischemic stroke is an inherent risk of CAG itself, besides arterial aneurysm at site of puncture may occur [7]. Thus, weighing the risks and benefits of CAG is challenging in AIS. The American Stroke guidelines on appropriate diagnostic steps remain vague [4]. Identification of factors that are associated with absence of obstructive CAD in AIS patients with evidence of myocardial injury would aid clinical decision making.

Therefore, we aimed to (1) describe frequency, indications, diagnostic findings, and therapeutic interventions of CAG in AIS patients, and (2) to determine clinical variables associated with absence of obstructive coronary artery disease.

## Methods

Patients were identified from two hospital databases (Charité-Universitätsmedizin Berlin, Germany, and Inselspital Bern, Switzerland). We searched for patients diagnosed with AIS who underwent CAG during the same hospital stay after the qualifying cerebral ischemic event (AIS) between 2011 and 2018. One author (SL) was responsible for the evaluation of the hospital data.

We excluded patients with stroke following CAG (i.e., stroke as complication of CAG). Diagnosis of AIS required cerebral imaging (MRI or CT) with exclusion of cerebral hemorrhage. Patients from the prospective TRELAS study (February 2011 to August 2013 in Berlin) were included [9].

In both hospitals, hs-TnT was assessed by the Roche Elecsys assay. The 99th percentile of the assay is at 14 ng/l with a limit of detection at 3 ng/l [10]. Obstructive coronary artery disease was defined as lumen diameter reduction ≥ 50% in one or more coronary artery vessels measured in CAG [11]. The ECG reports were assessed for signs of myocardial ischemia according to the 4th universal definition of myocardial infarction [7]. The presence of wall motion abnormalities derived from written reports and was either assessed in echocardiography or ventriculography part of CAG.

Cardiovascular risk factors (CVRF) were assessed in all patients. Published definitions for diabetes mellitus, hyperlipidemia and arterial hypertension were applied [12]. For every patient, the GRACE-, Killip-, CRUSADE- and HEART-score were calculated. The GRACE- and Killip-Score are prediction models for mortality in patients with ACS [13, 14]. The CRUSADE-Score stratifies bleeding risk in NSTEMI-patients [15]. The HEART-Score is a measure for risk stratification in the emergency department for patients with chest pain [16].

### Statistics

**Fig. 1:**
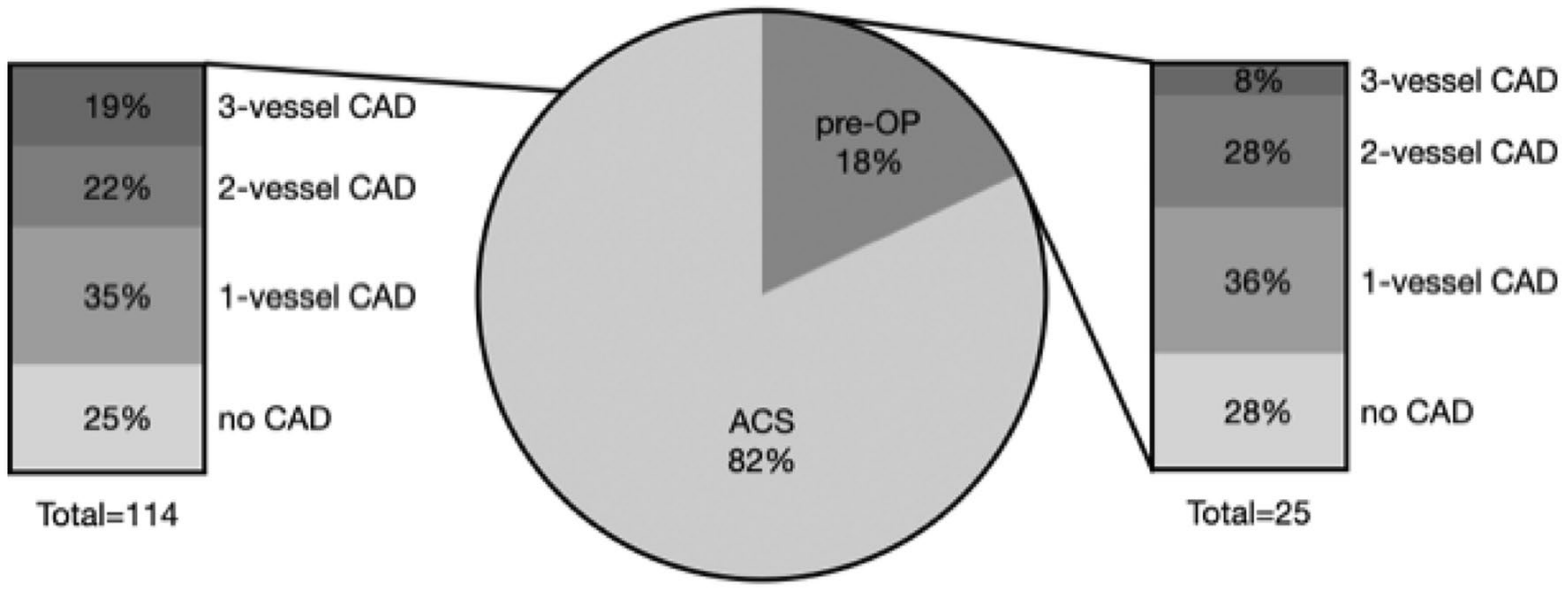
Indication for coronary angiogram and prevalence of CAD in 139 AIS patients. *CAD* coronary artery disease, *ACS* suspected acute coronary syndrome, *pre-OP* pre (cardiac) operation

We used descriptive statistics to present frequency, reasons, characteristics, and findings of CAG in stroke patients. We report mean plus standard deviation for continuous variables and median plus interquartile range for skewed data. We compared characteristics of categorical variables using the *χ*^2^ test and the *t* test or Mann–Whitney *U* test for continuous variables. A *p* value < 0.05 was considered statistically significant. Unadjusted odds ratios were calculated to assess the association between characteristics of stroke patients and the absence of coronary artery disease (CAD). Multiple logistic regression was applied to identify factors independently associated with the absence of CAD using stepwise backward selection approach. Variables were eliminated based on a *p* value threshold of 0.1 and the probability of the likelihood ratio. Positive and negative predictive values were calculated for absence of CAD. All analyses were performed using IBM SPSS Statistics 25 (Fig. 1).

## Results

From 2011 to 2018, we screened about 18,600 patients at two university hospitals (9000 patients in Bern and 9600 patients in Berlin) for the diagnosis of AIS and the intervention of CAG. We identified 139 AIS patients (median age 71 years, 68% male, see supplement material), in whom a subsequent CAG was performed. Hence, approximately 0.7% of patients admitted to the two participating centers (0.8% in Berlin and 0.7% in Bern) underwent CAG after stroke. The median time from stroke onset to start CAG was 4 days (IQR 2–9 days).

### Indications for coronary angiogram

CAG was performed either because of suspected acute coronary syndrome (*N* = 114; 82%) or for scheduled subsequent cardiac (valve) surgery (*N* = 25, 18%) or both (*N* = 3). Cardiac valve surgery was indicated due to endocarditis in 15/25 patients. Signs and symptoms that substantiated the clinical suspicion of ACS in AIS patients are reported in Table 1. Clinical symptoms of ACS (chest pain, dyspnea) were present in one in four patients (24%), abnormal laboratory findings were present in the vast majority of patients (hsTnT > 14 ng/l in 83%), ECG signs of myocardial ischemia (e.g., ST-elevation) in about one in three (36%) and wall motion abnormalities in half of the patients (56%).

**Table 1:** Signs and symptoms that prompted suspicion of ACS and led to coronary angiogram in 114 AIS patients

Characteristic	*n* (%)
Elevated hs-cTnT > 14 ng/l, *n* (%)	94 (83%)
Elevated hs-cTnT > 52 ng/l, *n* (%)	50 (44%)
Evidence of acute myocardial injury (hsTnT change > 20%), *n* (%)	53 (47%)
ECG signs of myocardial ischemia, *n* (%)	41 (36%)
Clinical symptoms (angina pectoris, dyspnea), *n* (%)	27 (24%)
Wall motion abnormalities, *n* (%)	64 (56%)
LVEF, median (IQR)	50% (34–65%)
Normal LVEF ≥ 65%, *n* (%)	29 (27%)
Moderately reduced LVEF 40–64%, *n* (%)	48 (44%)
Severely reduced LVEF < 40%, *n* (%)	32 (29%)

*ECG* electrocardiogram, *LVEF* left ventricular ejection fraction, *IQR* interquartile range

### Findings and therapeutic consequences of CAG in AIS patients

CAG findings in AIS patients are depicted in Table 2. CAD was demonstrated in approximately three out of four AIS patients (*n* = 105), and newly diagnosed in more than half (54%, *n* = 75). The prevalence of CAD did not differ between AIS patients with assumed ACS (76%) and those undergoing CAG in preparation of cardiac surgery (72%). Acute stenting of coronary arteries was prompted in one third of the AIS patients (37%, *n* = 51). Any kind of intervention (acute/staged stenting, bypass surgery) was performed or recommended in more than half of the AIS patients (61%, *n* = 84).

**Table 2:** Findings of coronary angiography in AIS patients (*n* = 139)

CAG finding	Suspected ACS (*n* = 114)	Pre-OP (*n* = 25)	*p* value
CAD (stenosis ≥ 50%), *n* (%)	87 (76%)	18 (72%)	0.649
1-vessel CAD, *n* (%)	39 (34%)	9 (36%)	0.865
2-vessel CAD, *n* (%)	24 (21%)	7 (28%)	0.450
3-vessel CAD, *n* (%)	24 (21%)	2 (8%)	0.130
CAG intervention (all), *n* (%)	75 (66%)	14 (56%)	0.365
Acute, *n* (%)	52 (46%)	4 (16%)	0.006
Staged, *n* (%)	10 (9%)	1 (4%)	0.432
Bypass, *n* (%)	13 (11%)	9 (36%)	0.002

*CAD* coronary artery disease, *CAG* coronary angiography, *ACS* acute coronary syndrome, *pre-OP* pre (cardiac) operation

### Variables associated with absence of CAD

The analysis of factors associated with absence of obstructive CAD was restricted to patients with suspected ACS (114 AIS patients). Out of 114 AIS patients with assumed ACS, 27 did not have obstructive CAD (24%) and 87 patients had evidence of CAD (76%). Group-comparisons and odds ratios are presented in Table 3.

**Table 3:** Predictors for absence of obstructive CAD in CAG in AIS patients with clinically suspected ACS

All (*n*) = 114	Missing values (*n*)	CAD absent *n* = 27 (24%)	CAD *n* = 87 (76%)	*p* value	Unadjusted OR (95% CI)
Female, % (*n*)	0	41% (11)	29% (25)	0.315	0.59 (0.24–1.44)
Age, years	0	68 (58 to 75)	75 (68 to 81)	0.008	0.95 (0.92–0.99)
Age ≥ 75 y, % (*n*)	0	19% (5)	47% (41)	0.008	0.26 (0.09–0.74)
Stroke characteristics					
NIHSS, median (IQR)	1	5 (2 to 11)	3 (1 to 6)	0.024	1.08 (1.01–1.16)
Ischemic insular cortex lesion, % (*n*)	0	44% (12)	13% (11)	0.001	5.53 (2.06–14.84)
Thrombolysis, % (*n*)	0	44% (12)	18% (16)	0.008	3.55 (1.40–9.02)
Lacunar vs non lacunar (TOAST), % (*n*)	1	4% (1)	3% (3)	0.950	1.06 (0.11–10.67)
Cardio embolic vs non-cardio embolic (TOAST), % (*n*)	1	49% (13)	40% (35)	0.467	1.38 (0.58–3.29)
Time onset to CAG in d, median (IQR)	0	3.7 (2.0 to 6.0)	3.9 (2.5 to 8.0)	0.305	1.02 (0.98–1.07)
Cardiac findings (non-invasive)					
WMA (echo/CAG), % (*n*)	1	35% (9)	63% (55)	0.010	0.31 (0.12–0.77)
LVEF in %, median (IQR)	5	63.5 (42.3 to 73.2)	50.0 (30.0 to 60.0)	0.016	1.03 (1.01–1.06)
LVEF ≥ 40%, % (*n*)	5	78% (21)	64% (56)	0.200	2.02 (0.69–5.95)
ECG, signs of ischemia, % (*n*)	0	26% (7)	39% (34)	0.213	0.55 (0.21–1.43)
Clinical symptoms, % (*n*)	2	7% (2)	29% (25)	0.033	0.19 (0.04–0.87)
Scores					
Killip-class, median (IQR)	1	1 (1 to 1)	1 (1 to 2)	0.066	0.27 (0.07–1.09)
GRACE-score, median (IQR)	9	99 (73 to 116)	118 (97 to 133)	0.022	0.98 (0.96–1.00)
CRUSADE-score, median (IQ R)	11	29 (25 to 35)	35 (24 to 45)	0.114	0.97 (0.93–1.01)
HEART-score, median (IQR)	9	4 (3 to 4)	5 (4 to 6)	0.001	0.61 (0.45–0.83)
Laboratory measurements					
hsTnT admission in ng/l, median (IQR)	5	51 (25 to 146)	43 (17 to 99)	0.666	1.00 (1.00–1.00)
Peak hsTnT (before CAG) in ng/l, median (IQR)	4	146 (56 to 513)	121 (40 to 417)	0.688	1.00 (1.00–1.00)
hsTnT dynamic change (before CAG) in %, median (IQR)	11	12.7 (− 6.3 to 235.9)	10.3 (− 6.7 to 129.8)	0.860	1.00 (1.00–1.00)
Peak CK in U/l, median (IQR)	6	139 (86 to 382)	177 (101 to 376)	0.766	1.00 (1.00–1.00)
NTproBNP in ng/l, median (IQR)	89	1459 (1196 to 3240)	3945 (814 to 7020)	0.860	1.00 (1.00–1.00)
CRP in mg/l, median (IQR)	1	4.2 (1.9 to 16.0)	5.0 (3.0 to 12.7)	0.687	1.00 (0.99–1.01)
HbA1c in %, median (IQR)	12	5.7 (5.4 to 6.0)	6.1 (5.7 to 6.8)	0.182	0.73 (0.46–1.16)
Cardiovascular risk factors					
Number of CVRF, median (IQR)	2	2 (1 to 2)	2 (2 to 3)	0.002	0.48 (0.30–0.76)
CVRF *n* > 2, % (*n*)	2	19% (5)	46% (40)	0.012	0.26 (0.09–0.74)
Hypertension, % (*n*)	0	67% (18)	86% (75)	0.026	0.32 (0.12–0.88)
Diabetes mellitus, % (*n*)	0	15% (4)	38% (33)	0.032	0.29 (0.09–0.90)
Smoking current, % (*n*)	0	22% (6)	43% (37)	0.063	0.39 (0.14–1.05)
Hyperlipidemia, % (*n*)	2	56% (15)	98% (85)	0.334	0.65 (0.27–1.56)
AF, % (*n*)	0	26% (7)	23% (20)	0.754	1.17 (0.43–3.17)
History of stroke, % (*n*)	0	11% (3)	26% (23)	0.109	0.35 (0.10–1.27)
Prior known CAD, % (*n*)	0	3.7% (1)	27.6% (24)	0.009	0.10 (0.01–0.79)
Previous medication					
Antiplatelet, % (*n*)	3	26% (7)	48% (44)	0.026	0.34 (0.13–0.90)
Oral anticoagulation, % (*n*)	3	4% (1)	12% (10)	0.237	0.30 (0.04–2.46)
Betablocker, % (*n*)	6	20% (5)	18% (16)	0.845	1.12 (0.36–3.45)
Statin, % (*n*)	3	23% (6)	38% (32)	0.171	0.50 (0.18–1.37)
Vital signs					
BPsys admission in mmHg, median (IQR)	9	143 (129 to 171)	155 (138 to 176)	0.461	0.99 (0.98–1.01)
BPdia admission in mmHg, median (IQR)	9	80 (62 to 93)	80 (71 to 97)	0.927	1.00 (0.98–1.02)
HR in bpm, median (IQR)	2	71 (71 to 76)	71 (71 to 89)	0.233	0.98 (0.95–1.01)

*CAD* coronary artery disease, *NIHSS* National Institutes of Health Stroke Scale, *CAG* coronary angiography, *d* days, *WMA* wall motion abnormalities, *LVEF* left ventricular ejection fraction, *ECG* electrocardiogram, *CVRF* cardiovascular risk factors, *AF* atrial fibrillation, *BPsys* systolic blood pressure, *BBdia* diastolic blood pressure, *HR* heart rate, *bpm* beats per minute

Clinical symptoms typical for ACS (7% versus 29%, *p* = 0.03) and wall motion abnormalities (33% versus 63%, *p* = 0.01) were less frequent in AIS patients with absence of obstructive CAD. Cardiac troponins did not differ between patients with and without obstructive CAD, neither (absolute) concentration levels nor their dynamic changes. Ischemic lesions in the insular cortex were strongly associated with the absence of CAD (unadjusted OR = 5.53, CI 95% 2.06–14.84). In addition, absence of cardiovascular risk factors was strongly associated with absence of CAD (arterial hypertension: unadjusted OR 0.32, 95% CI 0.12–0.88; diabetes mellitus: unadjusted OR 0.29, 95% CI 0.09–0.90). Both median GRACE (unadjusted OR 0.98, 95% CI 0.96–1.00) and HEART scores (unadjusted OR 0.61, 95% CI 0.45–0.83) were lower in patients without CAD (Table 4).

**Table 4:** Factors strongly associated with absence of CAD in multivariate analysis

	OR	Confidence interval (95%)	*β*	*p* value	Positive predictive value	Negative predictive value
Insular lesion present	7.23	1.59–32.92	1.98	0.011	52.2%	84.5%
No wall motion abnormalities	5.33	1.27–22.38	1.67	0.022	34.7%	85.9%
CVRF < 3	4.89	1.36–17.67	1.59	0.015	32.8%	88.9%
No clinical symptoms	6.90	0.78–60.87	1.93	0.082	29.4%	92.6%
Age ≤ 75 years	3.48	0.91–13.29	1.25	0.068	32.4%	89.4%

*CVRF* cardiovascular risk factors, *OR* odds ratio

In an exploratory multivariable (adjusted) regression model including variables associated with absence of CAD in univariable comparison (age ≤ 75 years, NIHSS, ischemic insular cortex lesion, presence of less than 3 CVRF, no wall motion abnormalities, ECG signs of myocardial ischemia, no clinical symptoms, thrombolysis, prior known CAD, antiplatelets) the strongest association was observed for ischemic insular cortex lesion, absence of wall motion abnormalities, less than 3 CVRF, age ≤ 75 years and absence of clinical symptoms. The Hosmer–Lemeshow test of the model is not significant (*p* = 0.984) indicating a good fit of the regression model.

## Discussion

In this retrospective analysis of consecutive AIS patients of two tertiary care hospitals, CAG was performed in less than 1% of all patients. The main reason to perform CAG was suspected ACS (82%) followed by scheduled cardiac surgery (18%). Obstructive CAD was absent in one of four patients undergoing CAG despite suspected ACS. Absence of clinical symptoms, absence of wall motion abnormalities (WMA), low number of CVRF, lower age and particularly lesions of the insular cortex were strongly associated with absence of obstructive CAD. These observations may help clinicians in decision making when weighing risks and benefits of CAG in AIS patients with suspected ACS.

The prevalence of CAD in our cohort of AIS patients undergoing CAG was high (74%). In more than half of the patients (53%), CAD was not known before. Amarenco et al. had found a lower prevalence of CAD (26%) in patients without prior known CAD but had performed CAG routinely [1]. Our numbers correspond to Scheitz et al. who had investigated AIS patients with elevated hs-cTnT and found CAD in 52% of patients [1, 17].

Evaluation of AIS patients with myocardial injury is a common clinical challenge [5]. The cardinal symptom of ACS (i.e., chest pain) may be absent in AIS patients due to due to sensory deficits, distorting body perception, or other neurological symptoms such as aphasia, anosognosia, or reduced level of consciousness. Myocardial infarction may, therefore, be missed in standard-of-care acute stroke workup. Underdiagnosis of myocardial infarction is known from patients with diabetes mellitus or older people [18, 19]. In the situation of AIS, myocardial injury may emerge from pathophysiologic pathways independent from ACS [20]. The concept of ‘Stroke Heart Syndrome’ describes mechanism of myocardial injury after stroke and implies both ischemic and non-ischemic pathways, including increased plaque vulnerability, autonomic dysfunction with tachy- and bradycardia and arrhythmias, changes in blood pressure and microcirculatory dysfunction [6, 20].

In the setting of AIS, the clinician needs to weigh the risks and benefits of CAG. The benefit would be the greater, the more likely obstructive CAD is causing either type 1 or type 2 myocardial infarction [7]. The risks of CAG may outweigh the benefits if absence of CAD is likely. With absence of obstructive CAD, alternative mechanisms appear to be more relevant for post-stroke myocardial injury. Our findings highlight that assessment of medical history (number of CVRF, age), clinical examination (chest pain, dyspnea) and investigation of heart function (wall motion) all help to evaluate the likelihood of obstructive CAD. Therefore, our observation may be interpreted as an appraisal of basic clinical assessment. Our results do not support a pivotal role of troponin measurement in predicting absence of CAD.

In recognition of a strong stroke heart interaction, stroke characteristics are of great interest evaluating the myocardial injury. The results of TRELAS study reveal that angiographic findings in AIS patients differ from patients presenting in the emergency room with chest pain [17]. A previous analysis had shown an association of the ischemic affection of right dorsal anterior insular cortex with a dynamic hs-cTnT elevation [21]. In our cohort, lesions of the insular cortex were strongly associated with no CAD (OR 5.54). Furthermore, we found more severe strokes with higher NIHSS in patients with absence of CAD (OR 1.08). Potentially, an ischemic affection of the insular cortex triggers pathophysiological mechanisms of myocardial injury irrespective of coronary stenosis. This finding emphasizes the current research, which tries to achieve a better understanding of the mechanisms of post-stroke myocardial injury [22].

Of note, there was an association between the treatment of thrombolysis and absence of CAD. Therefore, some hemodynamic relevant stenosis might have been dissolved by thrombolysis. The variable prior known CAD was not statistically associated with absence of CAD on CAG in our multivariate analysis. From a clinical point of view, it does not make sense to assume absence of CAD if CAD is already known from antecedent investigations of course.

### Limitations

This is a retrospective analysis of consecutive acute stroke patients from two hospital cohorts. Bias by selection and bias by indication apply. The high prevalence of CAD in comparison to previous studies suggests a highly selective cohort. The interdisciplinary decision to perform CAG was made in clinical routine and we cannot claim a standardized, uniform approach. Furthermore, there was no central reading of the coronary angiograms nor evaluation of the cardiological diagnostic (ECG, echocardiography). More standardized data will be provided by the prospective PRAISE trial [23].

Results may have been different in a case series not including application of intravenous thrombolysis.

## Conclusion

The decision to perform CAG in AIS patients is challenging. Absence of (1) clinical symptoms, (2) wall motion abnormalities, (3) vascular risk factors, or (4) older age and the presence of (5) ischemic insular cortex lesions make CAD in AIS patients less likely, even in the presence of myocardial injury. This may help clinicians in decision making.

## Supplementary Information

Below is the link to the electronic supplementary material.

Supplementary file1 (PDF 130 kb)

## Abbreviations

ACS: Acute coronary syndrome
AIS: Acute ischemic stroke
CAD: Coronary artery disease
CAG: Coronary angiography
cTn: Cardiac troponins
CVRF: Cardiovascular risk factors
hs-TnT: High-sensitivity cardiac troponin T
MI: Myocardial infarction
WMA: Wall motion abnormalities
