# Supplementary material for: Coronary angiography in acute ischemic stroke patients: frequency and determinants of pathological findings in a multicenter cohort study
Source: J Neurol. 2022 Feb 19;269(7):3745–51. doi: 10.1007/s00415-022-11001-5 (PMC9217821; doi:10.1007/s00415-022-11001-5)
Supplement: Supplementary file 1 — Supplementary file1 (PDF 130 kb) [file 415_2022_11001_MOESM1_ESM.pdf]

SUPPLEMENTAL MATERIAL

Coronary angiography in acute ischemic stroke patients: frequency and determinants of pathological findings in a multicenter cohort study

Supplementary Table 1.

Baseline characteristics of 139 patients with AIS that underwent CAG

**Characteristic**	**Value (n= 139)**
Age, years, median (IQR)	71 +- 12
Female, % (n)	31% (43)
NIHSS, median (IQR)	3 (1 – 6)
mRS, median (IQR)	3 (2 – 4)
Thrombolysis, % (n)	21% (29)
Time from onset to CAG in days, median (IQR)	4 (2 – 9)
**Cardiovascular risk factors**	
Diabetes mellitus, % (n)	29% (40)
Hypertension, % (n)	80% (111)
Hyperlipidemia, % (n)	60% (84)
Smoking (current), % (n)	36% (50)
**Medical history**	
Atrial fibrillation, % (n)	24% (27)
Previous stroke, % (n)	21% (29)
Previous MI, % (n)	14% (20)
History of CAD, % (n)	22% (30)
History of CHF, % (n)	35% (49)
History of COPD, % (n)	6% (8)
History of malignant tumor, % (n)	6% (8)
**Laboratory measures**	
hsTnT admission, median (IQR)	44ng/l (17ng/l – 124ng/l)
Hs-TnT, above URL, % (n)	77% (107)
CK admission, median (IQR)	106 U/l (61U/l – 176U/l)
Creatinine in mg/dl, median (IQR)	0.95 (0.82 – 1.16)
Renal insufficiency (Creatinin > 1,2mg/dl), % (n)	20% (27)
**Clinical assessment**	
Heart rate on admission in bpm, median (IQR)	71 (71 – 85)
BPsys on admission in mmHg, median (IQR)	152 (136 – 176)
BPdia on admission in mmHg, median (IQR)	80 (70 – 96)
Killip-class 1, % (n)<br>Killip-class 2, % (n)<br>Killip-class 3, % (n)	75% (107)<br>17% (24)<br>1% (1)
CRUSADE-Score, median (IQR)	32 (25 – 43)
GRACE-Score, median (IQR)	114 (93.5 – 129)

HEART-score, median (IQR)	5 (4 – 6)
**Medication history**	
Previous antiplatelet use, % (n)	42% (59)
Previous oral anticoagulation, % (n)	10% (14)
Previous statin use, % (n)	34% (47)
Previous use of β-blockers, % (n)	18% (25)

SD = standard deviation, IQR = interquartile range, NIHSS = National Institutes of Health Stroke Scale, mRs= modified Rankin scale, MI = myocardial infarction, CAD = coronary artery disease, CHF = chronic heart failure, COPD = chronic obstructive pulmonary disease, URL = upper reference limit, bpm = beats per minute, BPsys = systolic blood pressure, BPdia = diastolic blood pressure.
